# Implementation challenges of maternal health care in Ghana: the case of health care providers in the Tamale Metropolis

**DOI:** 10.1186/1472-6963-14-7

**Published:** 2014-01-06

**Authors:** Emmanuel Banchani, Eric Y Tenkorang

**Affiliations:** 1 Department of Sociology, Memorial University of Newfoundland, St. John’s A1C 5S7, Canada

**Keywords:** Implementation, Millennium Development goal, Maternal health, Ghana

## Abstract

**Background:**

Achieving the Millennium Development Goal (MDG) of improving maternal health has become a focus in recent times for the majority of countries in sub-Saharan Africa. Ghana’s maternal mortality is still high indicating that there are challenges in the provision of quality maternal health care at the facility level. This study examined the implementation challenges of maternal health care services in the Tamale Metropolis of Ghana.

**Methods:**

Purposive sampling was used to select study participants and qualitative strategies, including in-depth interviews, focus group discussions and review of documents employed for data collection. The study participants included midwives (24) and health managers (4) at the facility level.

**Results:**

The study revealed inadequate in-service training, limited knowledge of health policies by midwives, increased workload, risks of infection, low motivation, inadequate labour wards, problems with transportation, and difficulties in following the procurement act, among others as some of the challenges confronting the successful implementation of the MDGs targeting maternal and child health in the Tamale Metropolis.

**Conclusions:**

Implementation of maternal health interventions should take into consideration the environment or the context under which the interventions are implemented by health care providers to ensure they are successful. The study recommends involving midwives in the health policy development process to secure their support and commitment towards successful implementation of maternal health interventions.

## Background

Achieving the MDGs, especially improving maternal health has increasingly become the central focus of many multilateral and bilateral donor agencies. Although the developmental agenda emboldened in the MDGs address all countries of the world, sub-Saharan African countries (SSA) have the greatest challenges and stand to benefit most from the promotion of its principles, compared to other regions of the world. In comparison to the rest of the world, SSA countries have the highest rates of poverty and illiteracy, as well as the highest rates of child mortality, maternal mortality, HIIV/ AIDS and malaria [1].

By December 31, 2014, fourteen out of the fifteen years for reaching the targets and indicators of the MDGs would have passed. A relevant question is whether SSA countries can meet the targets and monitoring indicators within the stipulated time-frame, and if so, whether such an achievement can be sustained? If results would be achieved in Africa, there should be positive signs during the first decade of the millennium to point to this direction. Africa as a whole is off- track to meeting the MDGs on reducing child mortality and improving maternal health [2-5]. The number of maternal deaths has rather declined slowly in some SSA countries, with current trends indicating that Africa will not meet the target of reducing maternal deaths by 75 percent by the year 2015.

In Ghana however, it appears there are no enough studies on the challenges of implementing the MDGs with a focus on maternal health at the facility level [6,7]. We fill the void in the literature by examining the challenges of health care providers in their contribution towards improving maternal health in the Tamale Metropolis of Ghana.

Reducing maternal mortality and reaching the Millennium Development Goal to improve maternal health by 2015 appears to be very challenging in Ghana. A report by the Ghana Demographic and Health Survey (2008) indicates that the Maternal Mortality Ratio (MMR) has improved from 560 maternal deaths per 100,000 live births in 2003 to 451 maternal deaths per 100,000 live births in 2008. If the current trends continue, maternal mortality will reduce to only 340 per 100,000 by 2015, and it will be unlikely for Ghana to meet the MDG target of 185 per 100,000 by 2015 [6]. The high level of maternal mortality in the country in the midst of the numerous policies and interventions is a manifestation of what may be lacking in the implementation process. There is a multiplicity of factors involved in an attempt to explain these, but an examination of specific aspects of maternal health that pose a threat to the chances of women’s survival include issues of pre-natal and post-natal complications, unsafe abortion and other problems emanating from ignorance and lack of/or inadequate access to appropriate family planning programmes [8-10]. A look at the above causes of maternal health problems calls for among other things, an examination of how the nature and relationship between the availability, distribution and execution of skilled health providers and health services logistics have impacted the implementation challenges of maternal health care services, with the Tamale Metropolis as a case study. This has become more relevant especially as it has been observed that policy implementation remains one of the major problems confronting developing nations, including Ghana. In light of these arguments we will first examine the challenges confronting midwives in the implementation of maternal health interventions and policies and secondly, find out the challenges of maternal health logistics in health care facilities in the Tamale Metropolis.

### Study setting

The Tamale Metropolitan Area is one of the twenty (20) administrative districts in the Northern Region. It serves as Metropolitan and Regional capital. It is located in the centre of the region, approximately 175 km east of longitude 1°west and latitude 9° north. Savelugu/Nantong District bound it to the north, to the south by West and East Gonja, to the east by Yendi District, and to the west by Tolon/Kumbungu District. The Metropolis has a total population of 402,843 (projected at 2.9% regional growth rate from the 2000 census). The actual growth rate of the Metropolis is 3.5% which is higher than the regional and national growth rates of 2.9% and 2.8% respectively. It has a surface area of 1011 sq. km, which forms about 13% of the total land area of the Northern Region. Its population density stands at 384 persons per sq. km. In urban Tamale where there is ethnic diversity, Dagombas still constitute almost 80% of the total population. There are people from other regions and ethnicity in the metropolis. Almost all the people in Tamale rural areas are Dagombas. Islam is the predominant religion in the metropolis, with about 84% of the population affiliated to it. Christians constitute 13.6% (with Catholics forming 43.7%), traditional worshippers constitute 1.6%, and others form less than 1%.

## Methods

### Sample

The sample for the study comprised of twenty eight (28) participants from which information was solicited on the implementation challenges of maternal health care in the Tamale Metropolis (see Tables 1 and 2). The hospitals included the Tamale Teaching Hospital and the Tamale Central Hospital. These hospitals were selected because they are the main referral centers for health care services in the Metropolis. Moreover, considering financial and time constraints, it would have been impossible to cover all the health care facilities in the Metropolis. A more practical reason was that, considering the busy schedules of health professionals, it would have been difficult to get more participants in the study.

**Table 1:** In-depth interview with key informants

**No.**	**Position**	**Sex**	**Age**	**Years of experience**
1	Medical superintendent	M	52	21
2	Medical superintendent	M	49	16
3	Senior health services administrator	M	38	10
4	Health services administrator	M	31	4

**Table 2:** Participants in the focus group discussions

**FGD no.**	**Participants no.**	**Sex**	**Age (Years)**	**Profession**	**Years of professional experience**
1	1	F	57	Midwife	21
	2	F	52	Midwife	11
	3	F	48	Midwife	10
	4	F	54	Midwife	11
	5	F	48	Midwife	8
	6	F	53	Midwife	13
	7	F	52	Midwife	12
	8	F	52	Midwife	10
2	1	F	48	Midwife	12
	2	F	46	Midwife	11
	3	F	59	Midwife	23
	4	F	53	Midwife	16
	5	F	56	Midwife	13
	6	F	53	Midwife	15
	7	F	47	Midwife	12
	8	F	55	Midwife	17
3	1	F	47	Midwife	12
	2	F	51	Midwife	12
	3	F	54	Midwife	16
	4	F	51	Midwife	14
	5	F	49	Midwife	10
	6	F	55	Midwife	16
	7	F	54	Midwife	13
	8	F	49	Midwife	10

The study population in this study comprised of Midwives (24), Medical Superintendents [2] and Health Services Administrators [2] in two public hospitals in the Tamale Metropolis. The study used the purposive sampling technique to select study participants. The lists of practicing midwives were obtained from the Human Resource/Personnel Units of the hospitals from which the midwives were drawn to participate in the study. The selection criterion was midwives who had been practicing for over five (5) years mainly due to their experience in the implementation of maternal health interventions over the years. Those selected formed a group of eight participants each for a focus group discussion. Medical Superintendents and Health Services Administrators were also selected based on their experience, knowledge and involvement in the implementation of health programmes at the facility level.

### Data collection activities

The Focus Group Discussions (FGDs) were led by the researcher (first author) as the moderator who kept the conversations going, asked follow-up questions and sought clarification on issues that were not clear in the course of the discussions. A topic guide was developed to assist in collecting data from the FDGs. An assistant moderator trained by the researcher took notes and also operated the tape recorder. The groups were homogenous with respect to the profession. All the participants in the FGDs were females. In all, three (3) FGDs were held with midwives. The FGDs took place in the conference room and labour ward of the hospitals. Permission was sought from participants before recordings proceeded, although objections were raised by the participants for the fear that their voices might be heard on the radio. The researcher had to explain the purpose of the research was only for academic purposes. However, one group refused to be tape recorded. Each FGD lasted between forty five minutes (45) and an hour. FGD was used because it allows for the exchange of views and opinions through discussions with a group who are known to be concerned with, and knowledgeable about the issues discussed. Again, FGD is used to obtain knowledge, perspectives and attitudes of people about issues, and sought explanations for behaviours in a way that would be less easily accessible in response to direct questions [11].

In-depth interviews were held with health managers (Medical Superintendents and Health Services Administrators) who were the key informants. These interviews were conducted in the offices of the key informants and were tape- recorded, lasting between forty five (45) minutes in length and an hour, upon their permission. In all, four (4) in-depth interview sessions were held. The in-depth interviews sought information about the challenges in the implementation of maternal health interventions at the facility level, difficulties with the processes and guidelines in the implementation of such interventions, challenges with In-Service Training (IST) programmes, challenges of logistics for maternal health, training capacities of logistics personnel, challenges in the logistics management system, among others.

In-depth interview was used because it is useful in situations where either in-depth information is needed or little information is known about the area under discussion. Moreover, the flexibility allowed to the interviewer in what he or she asks of a respondent is an asset as it can elicit extremely rich information [12]. It also allows for intensive and systematic note-taking.

A variety of documents were collected as secondary sources of information. The documentary data were mainly from policy documents and annual reports of the Ministry of Health (MOH), Ghana Health Service (GHS), the Tamale Metropolitan Health Directorate annual reports and Annual Review Reports of the hospitals. These sources were to verify some of the information obtained from key informants and participants in the FGDs.

A semi-structured interview guide was developed for the study. Two sets of semi-structured interview guides were developed. The first set of semi-structured interview guide was for the midwives and the second set for Medical Superintendents and Health Service Administrators. The semi-structured interview guides had four main sections soliciting information on challenges of maternal health in the Tamale Metropolis. The first section dealt with issues about education and training, the second section focused on working conditions and maternal health logistics at the facility level, the third section dealt with implementation of maternal health policies, and the fourth, issues related to motivation in the profession of midwives such as salaries, promotion etc.

### Ethics

This study was approved by the Research Ethics Committee of Tamale Teaching Hospital and the Research Board of Tamale Central Hospital after formal letters for permission to conduct the study was sent to the management of the hospitals. All participants gave their informed consent to participate in the study after permission was granted. The study adheres to the RATS guidelines on qualitative research.

### Analysis

The data from the in-depth interviews, FGDs and field notes were transcribed verbatim. After transcription the data were organized into retrievable sections by assigning a code to each FGD and interview respectively using word-processing file. The researcher had to familiarize himself with the data by listening to the tape recordings from the FGDs and interviews over again to ensure that the appropriate transcription and coding were done. This was followed by preliminary coding data by identifying how the respondents conceptualized certain key phrases and words. The documents were analyzed using content analysis by identifying common themes that emerged taken into consideration the objectives of the study. The documents were sorted by types (annual reports and policy documents). From the various themes and categories that emerged, the data were analyzed thoroughly.

## Results

### Skilled birth attendants challenges

#### Inadequate In-Service Training (IST)

Participants in the focus group discussions stated that in-service trainings organized by the Ghana Health Service (GHS) were ineffective, and that the content of most of the IST programs did not address the scope of maternal health. Most of the IST programs took a long time to be organized, usually more than a year. Most midwives complained that this was affecting the quality of care of maternal health services in the metropolis. This is because they lack current practices and knowledge in topics such as family planning, breast-feeding, and infection prevention, among others. Also, it was reported by the midwives that the selection process for participation in such IST programs was unclear. Midwives reported that most of them were not aware of such training programmes, and by the time they became aware, participants for such programmes would have already been selected.

Since I started working in this facility for about four years now, I have never been part of any in-service training programme. For almost two (2) years now, none of us here have attended any IST. I am still relying on what I have learnt from school to attend to clients. Sometimes, you encounter difficult situations, especially management of the third stage of labour that you were not taught in school. You have to do try an error to save the mother and the child.

*What is even annoying is that, sometimes we hear of such training programmes from our colleagues and by the next moment the training programme is going on and we do not know the criteria they use to select people for such programes. Most of the time, it is our boss who attend such programmes only* (Midwife)*.*

### Limited knowledge of maternal health policies and guidelines

Majority of the midwives did not know the maternal health policies in the country and only a few were aware of the free maternal healthcare and the safe motherhood initiative. Others also heard of such policies from colleagues or from their immediate supervisors. It was also revealed in the FGD that most of the midwives did not know the content of the few policies and what specific issues they were addressing. Furthermore, several interpretations were assigned to the policies.

*I know that the free maternal health care covers only delivery and when pregnant women come here to deliver they are not charged anything. But I’m not sure if complications, antenatal and caesarean sections are covered* (Midwife)*.*

When asked about implementation guidelines for such policies, interview informants reported that they do not have any guidelines from the Ghana Health Service (GHS). They explained that there were directives from the GHS to implement such policies, but no policy guidelines were issued to that effect.

*As of now, we have not received any guidelines from the GHS and we are doing just as we have been told to do. It was a directive issued from the GHS to all public health care facilities in 2007 to start providing free services in the areas of antenatal care, normal delivery as well as child welfare services free of charge to women and their new born babies. The bills are forwarded to the NHIS for refund per the directives* (Medical Superintendent)*.*

### Workload and shortage of midwives

Workload was a major challenge in the provision of quality maternal health care services. Most of the midwives complained that they attended to more clients than usual and these reduced time spent with clients. They omit certain aspects of needed care for women resulting in incomplete medical examination. The midwives further explained that the workload was a result of the fact that their numbers were inadequate. There are shortages of midwives in the health facilities. As a result, one midwife had to attend to more clients than usual. The midwives had an overwhelming range of duties and responsibilities.

*We are just working here like we are machines. When you arrive for work in the morning, you will be on your feet till the evening. On the average one midwife has to attend to about 60 to 80 pregnant women in a day and you have to ignore certain aspects of care that are very crucial. You have to also perform other duties such as assisting doctors in obstetrics and gynecology cases, conducting normal vaginal delivery, keeping of records at the labour ward… and if I want to mention all, it will take us another two hours* (Midwife)*.*

### Risk of infections

The midwives complained that in the course of performing their duties, they are exposed to various risks of infections, including HIV/AIDS and Tuberculosis. This was mainly because the infection prevention items for delivery are inadequate or lacking. The midwives reported that there is high risk of infections discouraging most of them from performing deliveries. There were complaints about the risks of getting in contact with blood and body fluids during child birth. This has pulled away a number of midwives currently practicing in the area of delivery.

*This job is too risky. We come in contact with blood and other body fluids. In the process of performing our work like delivery and caesarian sections, some of our clients who have contracted deadly diseases like HIV/AIDS, tuberculosis and hepatitis B, which can easily be transmitted to you, especially when you do not wear protective gadgets* (Midwife)*.*

### Aging workforce

The retirement of aging midwives contributes to the diminishing cadre of practicing midwives. The majority of practising midwives were over fifty (50) years old in the facilities, and many would be retiring very soon. Also, most people were not interested in the profession and the intakes to midwifery training schools are limited.

*Over sixty percent (60%) of our midwives are over 50 years old. This year, seven of them will be retiring and this will have serious consequences on our workforce strength. We have been facing this problem every year, and we do not have authority to recruit midwives ourselves. It is the GHS who post midwives to our institution and we do not have control over that. For about two years now, no fresh graduate midwife has been posted to this facility* (Medical Superintendent)*.*

### Low motivation

The questions exploring motivation were centered on several themes, including salaries, promotion and transportation to work. It is believed that those engaged directly in implementing a policy should be motivated well enough to ensure the successful implementation of such a policy. The midwives complained that motivation in the profession was lacking. Most of the midwives agreed that their salaries were too meager and that they had not witnessed any increment in their salaries for a long time. The delay in the payment of salaries is also frequent and most of them had to find other ways of complementing their income.

*If you want to compare the work that we do in relation to our salaries, it is nothing to write home about. The salary is too small that it can not take you home considering the economic conditions in the country and the numerous responsibilities at your hands. For about four (4) years now, I have been receiving the same salary even though I know that from time to time there have to be an adjustment in salaries. There was even a time my salary stop coming for about three months. I have to make several complains to the authorities before it was finally restored. I do not even receive extra duty allowance for the time work that I do. The delay in the payment of salaries is also worrying. At the end of the month, it usually takes a week or more before your salary reflects in your account* (Midwife)*.*

Besides low salaries, delays in promotion were mentioned by the midwives as causing dissatisfaction in the profession. They believed the current system of promotion was not fair and usually took a long time, and at times not effected at all. There had to be follow-ups and in some cases bribes paid to officials to ensure they are promoted

*I will be going on retirement next year and I have applied for promotion since last year and up till now I have not heard anything from the authorities. When I decided to trace up I was given excuses here and there that they are still processing it* (Midwife)*.*

Personal transportation to work was also reported by the midwives. Most of them did not have personal means of transport to work and had to rely on public transport. As a result of this most of them came to work very late.

*Every day I have to pick a taxi to work which cost me GHC 3.00. I have to stand by the road side in order to board a taxi. Most of the cars that pass by are always full. You have to stand for a long time before you finally get one to board, by the time you get to work it is late and you are blamed for coming to work late* (Midwife)*.*

### Health logistics challenges

#### Limited equipment and supporting infrastructure

The environment under which most midwives operated was very appalling. Most of the midwives reported that there were no sufficient equipment like Manual Vacuum Aspirators (MVA), surgical gloves, wheel chairs, among others, to help them perform their duties successfully. This sometimes frustrated midwives.

*Most of our equipment are not in good shape at all. Most the wheel chairs are broken down, and this has made the movement of patients in and out of the operating theater very difficult for us. At times there are frustrations all over you, because when you go to the store room to request for surgical gloves, you are told the stocks have run out* (Midwife)*.*

The rooms under which they examined pregnant women are too small and do not have adequate ventilation.

*The room in which I examined pregnant women is too small. When you want to examine a pregnant woman, you have to pack your documents, table and seat to one corner of the room so that there will be enough space to lay the stretcher for the woman to lie down. After examining her, you arrange everything back and write your report. The room is also warm all day. As you can see, there is no ceiling fan or air conditioner to reduce the heat in the room. It is like you are working in an “oven”* (Midwife)*.*

Also, essential infrastructure like water and electricity are most of the times interrupted.

*The electricity and water supply systems are not reliable. There are usually intermittent power outages that usually put us in difficult situations, especially during caesarian sections, the power can go off without any prior notice and we do not have a stand by generator* (Midwife)*.*

Drugs needed to perform Basic Emergency Obstetric Care (BEmOC) are sporadically or completely unavailable.

### Inadequate ward space for delivery and resting

The in-patient wards for delivery are too small to accommodate a large number of pregnant women who come to deliver. Deliveries are done one at a time and pregnant women have to form a queue waiting for their turn. Sometimes, most of the pregnant women have to lie on the bare floor due to the fact that the delivery beds are not enough.

*You can see for yourself. The delivery beds are not enough so when we finished with a delivery case the mother and the child has to lie on the floor* (Midwife)*.*

### Problem with transportation

Inadequate and unreliable means of transport were mentioned by the key informants. The ambulances at the facilities served multiple purposes and were not be available at certain times. When patients are to be referred to another facility, the patient bore the cost of fueling the ambulance, which most patients are not able to afford. Most of the ambulances are over-aged.

*Our budget is insufficient to buy fuel for our ambulance and let alone purchase a new one. At the moment we have one ambulance that we use for the referral of pregnant women and other patients in complications we can not handle to other facilities. We are sometimes caught in the ‘web’ because we usually have multiple cases that we have to use one ambulance for all the cases one at a time* (Health Services Administrator)*.*

Moreover, trucks to convey maternal health logistics from the Regional Medical Store (RMS) to the health facilities are lacking. Most maternal health logistics are kept in RMS for a long time before they are finally delivered to the facilities.

*Currently we do not have trucks that will convey logistics from the RMS to the hospital. We sometimes use our official vehicles or hire trucks to convey the logistics to the facility* (Health Services Administrator)*.*

### Difficulties in following the procurement Act (Act 663, 2003)

Managers at the facilities level complained about the difficulties encountered when using the Public Procurement Act (Act 663, 2003) in the process of procuring essential commodities for maternal health. Key informant interviews showed that the Public Procurement Act (Act 663, 2003) did not allow for emergency purchases. Most of the times, there were shortages for essential commodities for maternal health in the facilities which require urgent replenishment, but because the procurement law had to be followed, it delayed the process of purchasing such commodities.

There are at times things are needed so urgently and you are being asked to follow the procurement law. If you do emergency purchase it is difficult to convince the auditors why there was such an emergency purchase and you have to do a lot of documentation to convince them.

*The Act is very strict. You can not procure without the requirements of the law. And the way the law is, it does not allow you to address some emergencies. If you require anything, the tender entity committee looks at it and approves it, then you go and invite bids, the bids come in, you open the bids in front of the public, and after that a committee is formed to evaluate the tenders, after evaluation, the tenders go to the tender entity committee for final approval… and a supplier is selected* (Health Services Administrator)*.*

### Weak supply chain for maternal health logistics

The facilities faced supply chain problems, ranging from forecasting, warehousing and storage, inventory control and logistics management information for maternal health.

Interviews with key informants revealed that staff at the logistics department had not received adequate training on the use of Microsoft Office Excel for forecasting. Also, the rate of consumption of maternal health logistics was not known in the facilities and as a result, they could not project future consumption levels.

Moreover, most of the storekeepers were not trained in basic storekeeping procedures. There were no order by which stocks were issued out like, the First Expired First Out (FEFO) method of issuing out stocks, and providing the appropriate temperature control for the logistics in the store room.

The feedback from user departments for maternal health logistics are not channeled to the stores department.

*The feedback from the user departments are not channeled well. When they run out of stocks, they do not report early for you to start the procurement process. The next thing is that they just come and tell you that they need this item urgently. We do not also have a monitoring and evaluation team that will make timely information for us to take decisions* (Health Services Administrator)*.*

## Discussion

The United Nations Millennium development Goal (MDG 5) aims to improve maternal health. This goal is structured around two key targets: First, to reduce maternal mortality rates by 75% between 1990 and 2015, and Second, to achieve universal coverage of skilled care at birth by 2015. Inequitable access to maternal health is a big challenge globally. There is also inequality of access to skilled care at delivery. The inequalities to maternal health are discussed with reference to the contextual factors as proposed by Walt and Gilson model [13].

As a useful starting point, the provision of adequate in-service training (IST) is considered vital in developing and keeping midwives with up to date practices in the field of maternal health care. The provision of adequate IST will go a long way to reduce maternal mortality. The clinical competencies of midwives need to be addressed through frequent IST and their curricular must have relevance in modern health care delivery practices. Consistent with our findings, several studies have also found that the clinical competencies of health providers in providing basic emergency obstetric care (BEmOC) are very low [14-16]. One of the major reasons why so many countries still have inadequate numbers of skilled midwifery providers is because those grappling with human resources have not paid attention to the need for 'proficiency' in the various competencies required to assist women and newborns. For too long it has been accepted that as long as the health workers received some (often too little) training in midwifery, this was sufficient [17]. There has to be clarity as to the understanding of competence- ability to perform aspects of the job and competencies, the basic knowledge skills and behaviours required of a midwife to practice safely in any setting [18].

The implementation of any policy requires that those involved in implementing such a policy have adequate knowledge of the policy. Those engaged in the implementation of a policy must be engaged in the formulation process of the policy. This will ensure the success of such a policy as their commitment and support will be high. Most of the policies in developing countries tend to be implemented through a top-down approach and are not communicated to those engaged in direct service delivery of health services as evinced in this study. In essence, the dissemination of maternal policies at the local level is weak. Communication is also an essential ingredient for the success of a policy. Failure to communicate a policy effectively may lead to implementation failure. It has been suggested that by specifying and providing clarity on the policy, and ensuring that the policy is transmitted to the appropriate personnel, should lead to successful implementation [19]. Besides, different meaning and interpretations assigned to the policy are minimal. In Thailand and the United States, most health professionals have low to moderate knowledge about the national policy, and their levels of involvement in policy formulation and implementation is low [20,21].

The low motivation of health professionals has contributed to the high exodus of health professionals out of the country, to international organizations and to the private sector. This has created shortages of midwives resulting in heavy workloads in health care facilities. Moreover, the low status and recognition accorded to midwives have discouraged people who want to pursue that profession. Marginalization of midwives also implies that they face feelings of disappointments and confusion [22] and may not be able to take their own initiatives. To be able to achieve the MDG 5 requires well motivated and dedicated midwives who will show commitment towards the delivery of quality maternal health services. Several studies have shown that the low motivation in the health sector has impeded the implementation of most policies and reforms in Ghana [23,24]. The low motivation of health professionals have forced some to supplement their income by engaging in other occupations.

Attention has focused recently on the importance of adequate and equitable provision of health personnel to raise levels of skilled attendance at delivery and thereby reduce maternal mortality [25-28]. However, the human resource crisis in health care means that many countries are far from reaching the health-related MDGs. Factors contributing to this crisis include mal-distribution and low workforce productivity together with an acute shortage of skilled workers in the government health sector [29]. The effects of shortage of health professionals for maternal health are reduction in quality of services; increased workload; reduced time for the patient; and poorer infection control [30]. When adequate skilled personnel are provided, they can better respond to the needs of maternal care, thereby, helping to reduce maternal mortality.

To enable midwives function effectively, there has to be an “enabling environment”. Provision of that environment for midwives will ensure delivery of quality maternal health care and reduction in maternal mortality. A skilled attendant should have the necessary equipment and medicines and adequate referral means to be effective in reducing maternal mortality. The environment can also be viewed broadly to include the political and policy context in which skilled attendants must operate, the socio-cultural influences, as well as the more proximate factors such as pre-and in-service training, supervision, deployment and health systems financing. This environment should also ensure there are sufficient skilled attendants with the necessary skills, satisfactory pay scales and career advancement opportunities; continuing education opportunities to maintain and upgrade skills; supportive supervision mechanisms; and possibilities of skilled attendants to refer women and newborns directly to higher level care if necessary [31].

Improving access to maternal health logistics is an essential component of strengthening maternal health programs and outcomes [32]. Maternal health challenges in the entire health system come with deeply embedded issues of human resources, infrastructure competing priorities and community engagement. The shortages of maternal health logistics serve as a direct barrier to the utilization and positive outcomes at health facilities [33,34]. Maternal health logistics often require a more highly trained health care provider who is available all the time. These providers are trained sufficiently on how to use these logistics. An efficient logistics system should be responsive to the needs of the end-users (the patients).

Improving logistics systems and ensuring product availability requires focusing on the customer regardless of the supply chain being considered [31]. A reliable and efficient transportation system should be key to the success of logistics systems and should be able to respond to emergencies and also ensure that products are in constant supply. A legal system that does not allow for easy access to logistics have implications on the way health logistics are procured, and could be detrimental to achieving quality maternal health care. Laws and legislations could impede the successful implementation of health policies, especially during the formulation of such policies, when provisions were not made to take consideration of such legislations. This should serve as a lesson to policy implementers of health interventions that, there is always the need to ensure that legislations are less rigid to allow for effective implementation of health interventions.

Furthermore, the logistics management system should effectively function in each of the components to ensure that there are no hindrances in handling maternal health logistics. In many developing countries, logistics systems for public health facilities have been centralized, with central ministry offices responsible for planning, forecasting, procurement, warehousing and the distribution of essential drugs, contraceptives and vaccines. These systems have been notoriously inefficient and in many cases incapable of providing adequate supplies on a timely basis [35].

In essence, the availability of resources is an important ingredient in ensuring the success of policy implementations. As reviewed earlier, without adequate resources, implementation of health policies would encounter challenges as resources to ensure the execution of such programmes are insufficient or lacking. Working on the systems that will ensure time delivery of logistics is crucial for achieving MDG 5.

## Conclusion

It can be drawn from the findings of the study that contextual factors affect the implementation of maternal health interventions by health care providers in the Tamale Metropolis. Implementation of maternal health interventions should take into consideration the environment under which the interventions are implemented by health care providers to ensure they are successful. The challenges emanating from maternal health care delivery in the health system indicate that human resources for health and logistics have a direct bearing and influence on the way maternal health interventions are implemented. To ensure that such interventions are successful, these issues need to be given priority in the implementation phase.

The findings also call for the provision of frequent IST to update midwives’ knowledge and skills so as to provide quality maternal health care. The top-down approach to policy implementation has contributed to the limited knowledge of maternal health policies among midwives and their support and contribution towards the success of such policies are minimal as revealed by participants in the study. The low motivation in the health sector has not been given priority, and most health policies have not factored this concern in the formulation of such policies. Without the requisite and adequate human resources in health, implementation of health policies will be a mirage stemming from a dissatisfied workforce that will migrate to other sectors where conditions of service are perceived to be better.

The absence of efficient logistics systems in health care facilities in the Tamale Metropolis has contributed to the challenges of delivering quality products to the final consumers that is patients. Without adequate supply systems, programmes and policies designed to reduce maternal mortality will not be achieved. The inability to have skilled personnel to manage the supply chain also means that products cannot be handled well and the quality of such products may be compromised and, therefore, may not lead to improvement in health of women their babies in the Tamale Metropolis.

### Lessons for policy and future research

A number of important lessons emerged from the findings of the study that should serve useful lessons to health policy implementers and policy analysts in developing countries. Unless these bottlenecks in the health system are considered, any attempts at achieving a reduction in maternal health may not be successful.

One important lesson that emerged out of the study findings is the top-down approach to policy implementation of health policies in Ghana. There is mostly little consultation between policy formulators and stakeholders at the local level who are expected to implement such policies. This has resulted in weak dissemination of policies for effective implementation. The policies are mostly formulated at the national level and may be disseminated to implementers at the local level without guidelines accompanying such policies, even though these guidelines may exist. The guidelines are mostly kept at the national level without conscious efforts to disseminate them. Local level implementers often use their own discretions, resulting in several meanings and interpretations assigned to the policy.

Moreover, most health policies have demonstrated a weak link towards addressing the paucity of human resources in health in the contents of the policies. These policies are designed most of the time, specifying how programmes are to be executed, but have failed to address the issues of motivated workforce and capacities of personnel driving the implementation of such policies.

Furthermore, most policies are implemented without the provision of adequate resources to ensure smooth implementation. Without adequate resources and infrastructure in place, health policy implementations in developing countries run into difficulties, because no consideration is given to the required infrastructure and resources. It is expected that future policy implementation would take into consideration the required infrastructure and resources before implementation proceeds.

It is also important to consider institutional bottlenecks that tend to hamper successful policy implementation. Most policies in developing countries often conflict existing laws and legislations, creating bureaucratic implementation. In so doing, the policies fail to achieve their intended purpose.

This study has some limitations that need to be acknowledged. Firstly, since the study was limited to two public hospitals, the findings may have limited generalisability to all health care providers in the Tamale Metropolis. Secondly the methodology of the study presents limitation in terms of small sample, especially the external validity, which refers to the extent to which the findings can be generalized. Further studies employing quantitative methods are needed so that the findings can be generalized, and make the study more replicable. Moreover, the study findings only consider implementation challenges faced by health care providers. Future research can explore the challenges faced by users in maternal health care (patients), especially at the household and community levels. Moreover, the study uses only contextual factors to assess health policy implementation. Future research can extend to the other variables such actors in health policy implementation, focusing on the challenges of Non-governmental Organisations (NGOs) in health. The study did not include other categories of skilled birth attendants such as Obstetricians and Gynecologists, thus the findings could not be assumed to apply to all skilled birth attendants in the Tamale Metropolis.

## Competing interests

The authors declare that they have no competing interests.

## Authors’ contributions

EB was involved in the study conceptualization and data collection, data analysis and drafting of the manuscript. EYT participated in the review of the literature, helped to critically revise the manuscript and edited the manuscript. All authors read and approved the final manuscript.

## Pre-publication history

The pre-publication history for this paper can be accessed here:

http://www.biomedcentral.com/1472-6963/14/7/prepub
